# 

**Published:** 1873-12

**Authors:** 


Vol. XIII.] DECEMBER, 1873. [No. 5.

SURG. GENL'S OFFICE. LIBRARY.

### BUFFALO

### Medical and Surgical Journal.

EDITED BY JULIUS F. MINER, M. D.

Professor of Special Surgery in the Buffalo Medical College ; Surgeon to the Buffalo General Hospital ; Surgeon to Buffalo Hospital of the Sisters of Charity, etc., etc.

BUFFALO.
PUBLISHED FOR THE EDITOR.

1873.

